# High density linkage disequilibrium maps of chromosome 14 in Holstein and Angus cattle

**DOI:** 10.1186/1471-2156-9-45

**Published:** 2008-07-08

**Authors:** Elisa Marques, Robert D Schnabel, Paul Stothard, Davood Kolbehdari, Zhiquan Wang, Jeremy F Taylor, Stephen S Moore

**Affiliations:** 1 Department of Agricultural, Food and Nutritional Science, University of Alberta, Edmonton, AB, T6G 2P5, Canada; 2 Division of Animal Science, University of Missouri, Columbia, Missouri, 65211, USA

## Abstract

**Background:**

Linkage disequilibrium (LD) maps can provide a wealth of information on specific marker-phenotype relationships, especially in areas of the genome where positional candidate genes with similar functions are located. A recently published high resolution radiation hybrid map of bovine chromosome 14 (BTA14) together with the bovine physical map have enabled the creation of more accurate LD maps for BTA14 in both dairy and beef cattle.

**Results:**

Over 500 Single Nucleotide Polymorphism (SNP) markers from both Angus and Holstein animals had their phased haplotypes estimated using GENOPROB and their pairwise r^2 ^values compared. For both breeds, results showed that average LD extends at moderate levels up to 100 kilo base pairs (kbp) and falls to background levels after 500 kbp. Haplotype block structure analysis using HAPLOVIEW under the four gamete rule identified 122 haplotype blocks for both Angus and Holstein. In addition, SNP tagging analysis identified 410 SNPs and 420 SNPs in Holstein and Angus, respectively, for future whole genome association studies on BTA14. Correlation analysis for marker pairs common to these two breeds confirmed that there are no substantial correlations between r-values at distances over 10 kbp. Comparison of extended haplotype homozygosity (EHH), which calculates the LD decay away from a core haplotype, shows that in Holstein there is long range LD decay away from the *DGAT1 *region consistent with the selection for milk fat % in this population. Comparison of EHH values for Angus in the same region shows very little long range LD.

**Conclusion:**

Overall, the results presented here can be applied in future single or haplotype association analysis for both populations, aiding in confirming or excluding potential polymorphisms as causative mutations, especially around Quantitative Trait Loci regions. In addition, knowledge of specific LD information among markers will aid the research community in selecting appropriate markers for whole genome association studies.

## Background

In previous studies, large variations in linkage disequilibrium (LD) have been reported [1-5]. Different measures of LD such as r^2 ^and D' are known to yield different conclusions in terms of the extent of LD. In studies using microsatellites and D' as a primary measure of LD [1-3] it was reported that LD extended for several megabases. On the other hand, when r^2 ^was used, LD was shown to be at background levels (r^2 ^at approximately 0.1) after only 500 kilo bases pairs (kbp) [4,5]. Differences in marker types used in these studies are also potential causes for LD variation, with microsatellites being more suitable for detecting long range LD than SNPs [6].

High resolution LD maps can provide information on specific markers that are part of haplotype blocks used in association analysis [4,7]. Previous whole genome linkage disequilibrium maps in cattle [5,7] have been used to analyze different aspects of LD. In the case of McKay *et al*. [5], approximately 3,000 markers (microsatellites and SNPs) were used to assess the extent of LD in eight different cattle breeds, while Khatkar *et al*. [7] analyzed the haplotype block diversity in Holstein-Friesan cattle using approximately 15,000 SNPs. The latter also used the Btau_3.1 build to arrange markers along the genome, however it is now known that BTAu_3.1 build has inconsistencies with other independently built cattle maps [8,9].

In addition, such LD maps can be considered a crucial tool for researchers looking to confirm or exclude potential polymorphisms as causative mutations. Recent studies using breed specific LD information have shed light on the importance of using LD information to link potential markers to economically relevant traits in cattle. In 2007, Olsen *et al. *[10] reported that a mutation in *ABCG2*, a gene responsible for secreting important substrates into milk [11], is the most likely candidate for affecting the observed milk yield quantitative trait loci (QTL) on BTA6 [12]. The approach used included constructing a dense marker map spanning the QTL region and using linkage and linkage disequilibrium information to assess polymorphisms in *ABCG2 *and other genes.

Correct marker order is crucial for construction of linkage disequilibrium and haplotype maps, as well as for future candidate gene searches on chromosomes harboring economically important traits. Bovine chromosome 14 (BTA14) is widely known to harbor quantitative trait nucleotides (QTN) with large effect on milk fat percentage [13] and marbling [14]. In addition, several QTL affecting other economically important traits have been identified on BTA14 [15,16].

This study focuses on the comparison of linkage disequilibrium (r^2^) between Holstein and Angus cattle using over 500 BTA14 single nucleotide polymorphism (SNP) markers on 331 Holstein and 137 Angus animals. As well, it identifies specific haplotype blocks and tagged SNPs for BTA14 which will be useful for future whole genome association studies.

## Results and discussion

**Figure 1:**
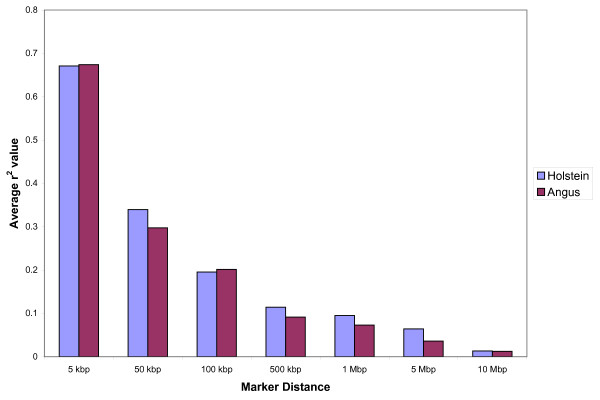
**Bovine chromosome 14 (BTA14) marker detail**. Average r^2 ^value for different marker distances (kbp) using 509 SNPs on Angus and 502 SNPs on Holstein animals.

Markers were binned according to marker distances (kbp) and r^2 ^was averaged and plotted for each category (Figure 1). LD drops from an average of 0.687 for Holstein and 0.648 for Angus to 0.328 and 0.317, respectively, when going from 1 kbp to 50 kbp marker distance in both breeds. Moderate levels of LD (r^2 ^at approximately 0.2) are reached at around 100 kbp and background levels (r^2 ^at approximately 0.1) at around 500 kbp. Both breeds show an inverse relationship between LD and marker distance, confirming recent studies on r^2 ^measures in cattle [4,5]. The average r^2 ^value for Holstein in McKay *et al. *[5] was higher (0.91) than in our study (0.687) for the 1 kbp inter-marker distance. This difference in value can be attributed to the wide range (0.005 to 1) in LD in our study (Additional file 1). McKay *et al*. [5] calculated LD using 81 markers for BTA14 compared to 502 in our study. The range in LD in our study is most likely a result of sampling of gametes to form successive generations [17] which is dependent on finite population size and not so much on the sample size. In this case, there could have been ancestral recombination between certain markers in close proximity, but not others. This is plausible, in the case of maternal haplotypes, when one considers the complexity of the pedigrees for both populations, with dams sometimes contributing information to multiple families. Another important aspect to mention in this analysis is the half-sib relationship among some dams in the Holstein population, causing slightly inflated LD values. In addition to these findings, there is a more rapid decline in LD for Angus compared to Holstein overall. Differences in effective population sizes for both breeds are a plausible explanation for this observed difference.

**Figure 2:**
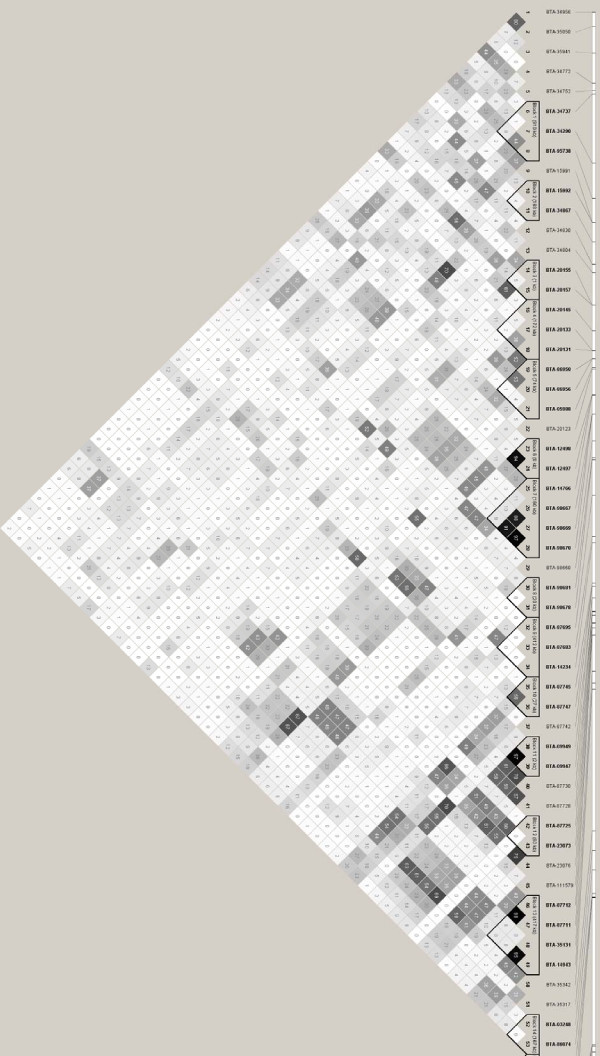
**Linkage disequilibrium (LD) map for Holstein cattle**. LD map of 502 SNP markers on Holstein cattle created using HAPLOVIEW [24]. For legibility purposes, only the first 53 markers are represented. The dark squares represent high r^2 ^values and triangles surrounding markers represent haplotype blocks under the four gamete rule [23]. A complete list of haplotype blocks is in Additional file 2.

**Figure 3:**
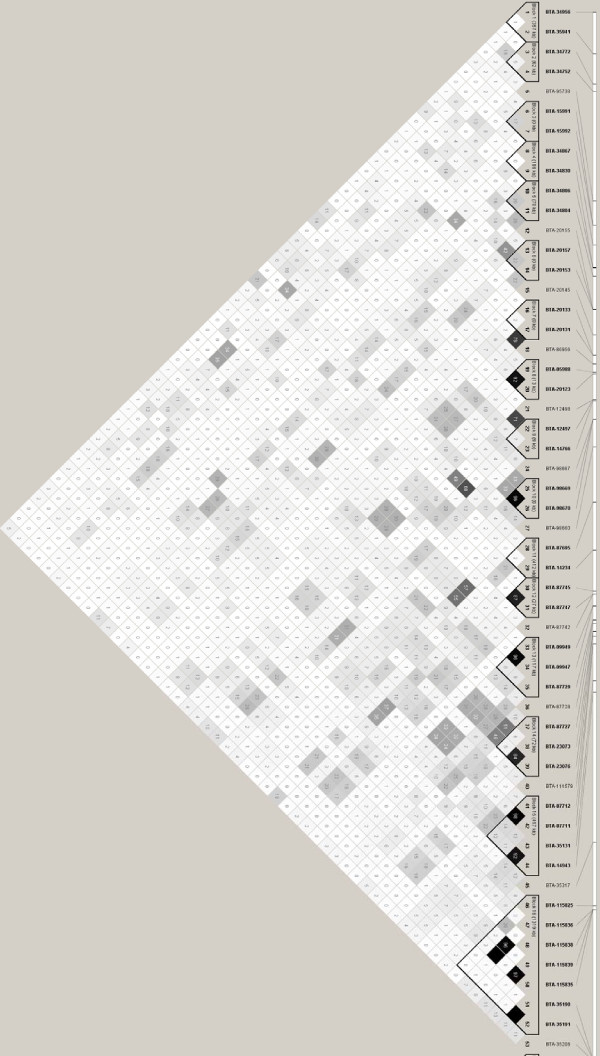
**Linkage disequilibrium (LD) map for Angus cattle**. LD map of 509 SNP markers on Angus cattle created using HAPLOVIEW [24]. For legibility purposes only the first 53 markers are represented. Dark squares represent high r^2 ^values and triangles surrounding markers represent haplotype blocks under the four gamete rule [23]. A complete list of haplotype blocks is in Additional file 2.

There are a number of algorithms used to define haplotype blocks [18-21]. The confidence interval algorithm [19] used by Khatkar *et al. *[7] relies on D' measures between markers to define blocks. The other approach used in LD analysis in dogs [22] and more recently in cattle [4] utilizes the four gamete rule [23] which defines blocks based on all 4 possible two-marker haplotypes existing with observed frequencies of at least 0.01. Using this method incorporated in HAPLOVIEW [24], 122 blocks (33 bp to 1338 kbp) were identified in Holstein and 122 blocks (45 bp to 1767 kbp) were identified in Angus (Figure 2, 3 and Additional file 2). The confidence interval method used by Khatkar *et al. *[7] found 27 blocks for BTA14. When the D' method was applied to our data, 64 blocks (33 bp to 1126 kbp) were identified in Holstein and 47 blocks (45 bp to 815 kbp) were identified in Angus (data not shown). Khatkar *et al. *[7] included 303 BTA14 markers on Holstein-Friesan cattle compared to 502 BTA14 markers in this study, so it is expected that as the number of markers increases more haplotype blocks are identified [25]. However, Khatkar *et al*. [7] not only used a different haplotype finding method, but also a different marker order causing differences in the number of blocks found. Another difference to take into consideration is that our haplotype block evaluation did not focus on coding regions, unlike Khatkar *et al*. [7]. Indeed, knowledge of LD within candidate genes is important, however non-coding elements such as miRNAs might also play a role in many inherited traits [26].

**Figure 4:**
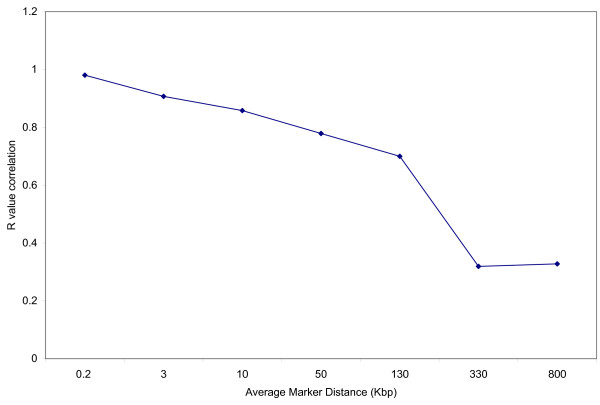
**Graph depicting the correlation of r-value for Holstein and Angus cattle**. Correlation of r-values between Holstein and Angus using 419 markers genotyped on both breeds. Values are plotted against average marker distances (kbp). R-values are presented in Additional file 3.

It is important to note that even though the extent of LD between these two breeds is similar, implementation of marker assisted selection based on the information from one breed cannot always be used for the other. In some cases, two markers at the same distance can show similar r^2 ^values in different breeds, but can be in different LD phase. For example: BTA-113824 and BTA-113826 have r^2 ^value of 0.988 in Holstein and 0.923 for Angus (Additional file 1). In order to verify if the same phase of LD between markers persisted for both breeds, the correlation of r values was calculated including all the same markers genotyped on both breeds. In order for markers to be in the same LD phase in both breeds, the r statistic has to be the same (value and sign) in both breeds [27]. Correlation of r statistic between Holstein and Angus indicates that a high correlation persists up to 10 kbp (Figure 4 and Additional file 3), agreeing with results from Goddard *et al*. [27]. This is not surprising since LD phase is less likely to be preserved between different breeds for longer distances. Therefore, careful examination of linkage disequilibrium measurement is necessary before applying genomic selection using the same SNP markers across these breeds.

Identification of haplotype blocks can be very useful in planning for association studies. The idea of selecting the minimum number of SNPs that define a particular haplotype of interest has been widely used in human genetics [21,24,28-31]. Together, haplotype blocks and SNP tagging focus on reducing the number of SNPs required for future association studies; thereby decreasing the cost associated with genotypes without the loss of precision in those studies. Using the tagger option [32] incorporated in HAPLOVIEW, 410 SNP markers were tagged in Holstein and 420 in Angus (Additional file 1). Briefly, this procedure defines a threshold for r^2 ^(default: 0.8) and SNPs tagged have LD measure higher than the threshold set. Of the total number tagged, 304 markers are common to both breeds. Using this approach, Hayes *et al. *[33] identified sites of preferential recombination when evaluating SNPs in four casein genes in goat milk. They were able to tag 11 SNPs that form part of different haplotypes, thereby reducing the cost of haplotype assisted selection (HAS) while identifying specific haplotypes associated with protein and fat percentage as well as milk volume.

Minor allele frequencies (MAFs) plotted against marker distances were used to observe any trends in decreased MAF. Such regions can indicate areas where alleles are reaching fixation, possibly because of selective pressure. In Holstein, acyl-CoA:diacylglycerol acyltransferase 1 (*DGAT1*) lysine variant has been increasingly selected for in this breed [13] due to its association with increased milk fat %. This frequency can vary between populations depending on the breeding goal implemented (high or low milk fat %) [34]. Using human coordinates from Marques *et al*. [8], the region between SNPs BTA-35050 and BTA-35941 were shown to be flanking the location of *DGAT1*. Calculation MAFs in this region showed an average MAF equal to 0.43 (Additional file 1). When analyzing nearby regions, a small cluster of low MAF SNPs is observed 7400 kbp away (Additional File 4). Considering our average estimate of LD reaching background levels (r^2 ^at approximately 0.1) at 500 kbp inter-marker distance, it is unlikely that these particular SNPs are in high LD with alleles from *DGAT1*; thereby implying that a higher density set of markers is needed in this region in order to make conclusions regarding the allele frequency trends around *DGAT1*. Screening the Angus breed for obvious signs of low MAF, approximately at the 30 Mbp region showed a cluster of low MAF SNPs (Additional file 5). These SNPs are located approximately 0.5 Mbp from a region of BTA14 where a carcass weight QTL has been detected [15].

In order to evaluate and compare the extent of LD for a candidate region between both Holstein and Angus animals, the extended haplotype homozygosity (EHH) approach [35] was used. Analyzing the extent of LD decay at various distances away from a specific candidate region can give insights into the selection histories of populations [36]. Basically, the EHH of an unselected allele increased to a specific frequency under neutrality will be different from the EHH of a selected allele raised to the same frequency under selection pressure. The method analyses the relationship between the allele's frequency and the extent of linkage disequilibrium surrounding it. A similar approach has recently been used in studies of signatures of selection in humans population, looking for candidate genes involved in different local adaptations [37]. Haplotypes with long range LD and with high frequency signify a recent positive selection or population bottlenecks [35]. The challenge is to determine whether the signature is due to selection or effects of population demography [36]. However, regardless of the LD causes, estimating and analyzing LD within a candidate region using appropriate algorithms can indicate selection on genes within this particular region. In general, segments of the chromosome where selected alleles are located will increase in frequency in a specific population as these selected alleles are pressured to reach fixation.

**Figure 5:**
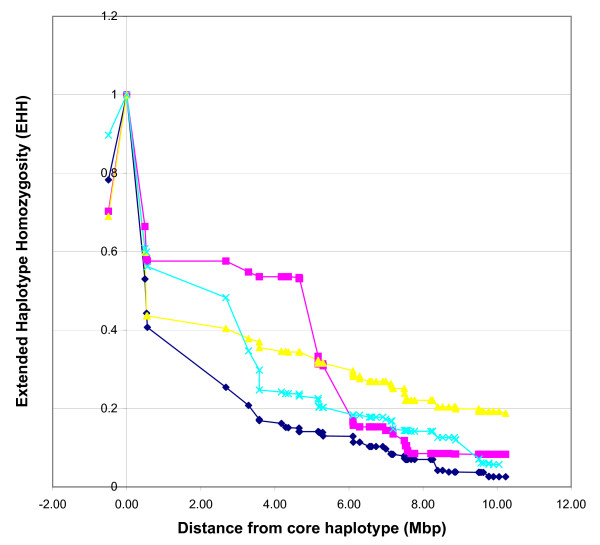
**Extended haplotype homozygosity (EHH) graph for Holstein cattle**. EHH values in Holstein evaluating the decay of LD on either side of the core haplotypes. Values plotted as a function of increasing marker distance. Markers BTA-35050 and BTA-35941 were used to make up the candidate region near acyl-CoA:diacylglycerol acyltransferase 1 (*DGAT1*). Marker positions are represented in mega base pair (Mbp). The pink plot represents haplotype AC with 15.1% frequency. The blue plot represents haplotype GA with 16.9% frequency. The yellow plot represents haplotype AA with 33.7% frequency. The light blue plot represents haplotype GC with 34.3% frequency.

**Figure 6:**
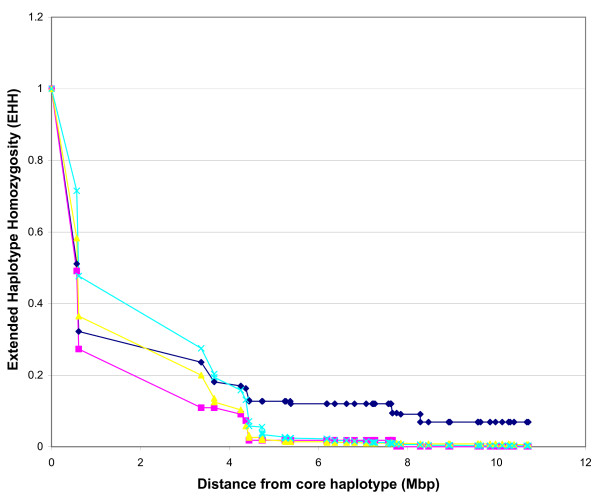
**Extended haplotype homozygosity (EHH) graph for Angus cattle**. EHH values in Angus evaluating the decay of LD on either side of the core haplotypes. Values are plotted as a function of increasing marker distance on Angus cattle. Markers BTA-34956 and BTA-35941 were used to make up the candidate region near acyl-CoA:diacylglycerol acyltransferase 1 (*DGAT1*). Marker positions are represented in mega base pair (Mbp). The pink plot represents haplotype CC with 6.41% frequency. The blue plot represents haplotype CA with 13.9% frequency. The yellow plot represents haplotype AC with 18.5% frequency. The light blue plot represents haplotype AA with 61.3% frequency.

In our analysis, the *DGAT1 *region was selected for EHH analysis and for comparison between the two breeds. In Holsteins, the second highest frequency haplotype, 33.3% (AA) showed the highest EHH when plotted up to 10 Mbp from the candidate region (Figure 5). Another haplotype, AC with a frequency of 15.1%, showed steady EHH values up until approximately 4 Mbp from the candidate region and consistently declined reaching EHH values under the AA haplotype. Within this same region, approximately 1.5 Mbp from *DGAT1*, lies *CYP11B1*, another gene linked to milk production traits in dairy cattle [38]. EHH analysis on Angus using SNPs in the same region showed little extended LD away from the candidate region (Figure 6). Haplotype AA, with frequency of 61.3%, showed declining EHH values after approximately 600 kbp away from the candidate region. EHH plots can be used to evaluate not only potential regions showing extended long range LD, but also long range LD between two gene variants, as shown with *DGAT1 *in Grisart *et al. *[13]. In this case, EHH values for the fat increasing haplotype (lysine allele) was consistently higher than for the alanine variant.

## Conclusion

Marker-phenotype association analysis coupled with information from bovine breed specific LD maps will be crucial for the research community, especially for chromosomes with extensive QTL information. Multi-locus LD can also aid in identifying specific haplotypes demonstrating long range LD decay and in determining regions in the genome where increased selection has occurred, even if the functional selection target is not known.

The increased availability of markers along with information from independently built maps will be a great aid in constructing accurate high density linkage disequilibrium and haplotype maps for a number of cattle breeds. LD information presented here should aid the community in future analysis for both Holstein and Angus breeds, possibly confirming or excluding potential polymorphisms as causative mutations, as well as increasing the power of QTL detection by selecting markers across BTA14 with specific amount of LD.

## Methods

### Animal Resource

Three hundred and thirty-one Holstein bulls provided by Semex Canada and one hundred and thirty seven American Angus bulls were used in this study. The Holstein bulls represent an eight generation extended pedigree. Angus families were selected to consist of one grandparent, one parent and three or more progeny. This pedigree structure has previously produced efficient estimates of phased haplotypes. Pedigree information for Holstein animals was obtained from the Animal Improvement Program Laboratory of the USDA [39]. Pedigree information for Angus bulls was provided by the American Angus Association [40].

### Selection and Genotyping of Markers

Single Nucleotide Polymorphisms (SNPs) included in this study were selected from the Bovine genome project [41] previously mapped onto BTA14 according to procedures described by Marques *et al. *[8]. SNPs were analyzed using an Illumina BeadStation 5.2 genotyping instrument (Illumina, Inc) and SNP genotypes were assigned using BeadStudio (Illumina, Inc) software.

### LD Analysis

Only markers successfully mapped on BTA14 were used in this study even if they were successfully genotyped on both breeds. Initially all 843 markers from Marques *et al. *[8] were genotyped. Thirty-one did not successfully amplify on both breeds. These markers were then filtered to exclude loci with a Minor Allele Frequency (MAF < 0.02 or that had greater than 10% missing genotypes within a breed This filtering resulted in 518 and 505 candidate loci in Angus and Holstein respectively which were used for further analysis. Genotype quality and haplotypes were estimated with GENOPROB 2.0 [42,43] using the map coordinates of Marques *et al. *[8] and the extended pedigree relating all animals within each breed. GENOPROB estimates the probability that a genotype is correct (pGmx) as well as identifies the most likely phase relationship between the alleles. Only high probability (pGmx ≥ 0.95) genotypes were considered for further analysis with no restriction used for order probability. Recent reports on GENOPROB showed that Holstein and Angus breeds produced the most accurately estimated genotypes and phased chromosome due to their complex pedigree structure [5]. Once paternal and maternal haplotypes were estimated they were inserted onto HAPLOVIEW [24] to verify their quality. The settings used included: min genotyped %: 50, Hardy Weinberg (HW) p-value cutoff: 0.0010, Minimum minor allele freq: 0.0010, Maximum # Mendel errors: 1. Overall, 9 markers dropped out from the Angus genotypes and 3 from Holstein. Markers passing the above filtering criteria were used to estimate LD using only the maternal haplotype and the program HAPLOXT [44]. The average marker spacing using this subset of markers was approximately 170 kbp, with the smallest and largest gaps between markers being 0.03 kbp and 2256.72 kbp, respectively. Maternal haplotypes were used in order to avoid biasing the linkage disequilibrium values due to the pedigree structure, which were solely paternal lineages.

Marker positions were inferred using the marker order from the 12 K RH map of Marques *et al. *[8] which is in high agreement with the recently released physical map based on the independent whole genome map of Snelling *et al*. [9]. Preliminary comparison between our marker order and the recently published bovine sequence assembly Btau_4.0 [41] shows agreement for markers common (737 markers) to both maps. Relative bp positions were calculated by dividing the highest centiray (cR) position by the corresponding bp position in the bovine sequence assembly Btau_3.1. The resultant average was approximately 17 kbp per cR. In regions where multiple markers had the same cR position, the sequence assembly distance was used. These closely mapped markers were in agreement with the assembly, according to results presented by Marques *et al*. [8].

Correlation of r-value used 419 markers common to both breeds. The r-value was calculated according to the formula [45]:

$$r=\frac{\left(freq(A1\_B1)*freq(A2\_B2)-freq(A1\_B2)*freq(A2\_B1)\right)}{\sqrt{freq(A1)*freq(B1)*freq(A2)*freq(B2)}}$$

Where A1 is the first allele of the first marker making up the haplotypes A1_B1 or A1_B2, A2 is the second allele of the first marker, B1 is the first allele of the second marker and B2 is the second allele of the second marker. Marker phase analysis was performed as follows: First, marker pairs had their r-values and inter-marker distances calculated. Next, the correlation of r-values (same marker pair) for each breed was calculated. Markers were then binned according to their inter-marker distance (category) and their correlation results averaged for each category. Haplotype and allele frequencies were calculated using SAS version 1.1.3 (SAS, Inc). Additional file 3 provides a list of markers and their calculated r-value for both breeds in each marker distance category.

Calculation of extended haplotype homozygosity (EHH) was performed using a EHH calculation web tool [46] designed according to procedures described by Sabeti *et al. *[35]. Only maternal haplotypes were loaded.

## Authors' contributions

EM collected the genotypes, analyzed the data and drafted the manuscript. RDS phased the genotypes and provided intellectual support. PS aligned sequences against the bovine sequence assembly. DK participated in the data extraction. ZW compiled SNP markers and designed genotyping assay. JFT provided Angus samples and SSM initiated and supervised the project. All authors read and approved the final manuscript.

## Supplementary Material

Additional file 1 **List of markers used for Linkage Disequilibrium (LD) analysis on Holstein and Angus cattle**. File includes: NCBI accession number, 12 K RH positions [8], inferred kilo base (kbp) positions as described in the materials and methods section, pairwise r^2 ^data, tagged SNPs and minor allele frequencies (MAFs). Click here for file

Additional file 2 List of haplotype blocks obtained in HAPLOVIEW [24] under the four gamete rule [23] for Holstein and Angus cattle. Click here for file

Additional file 3 Correlation of r-statistic using 419 markers genotyped on both Holstein and Angus cattle. Click here for file

Additional file 4 **Minor Allele Frequency (MAF) for 502 SNPs genotyped on Holstein**. MAFs were plotted against marker positions (kbp) on bovine chromosome 14. Red circle depicts the position of acyl-CoA:diacylglycerol acyltransferase 1 (*DGAT1*). Click here for file

Additional file 5 **Minor Allele Frequency (MAF) for 509 SNPs genotyped on Angus**. MAFs were plotted against marker positions (kbp) on bovine chromosome 14. Red circle depicts the position of acyl-CoA:diacylglycerol acyltransferase 1 (*DGAT1*). Orange circle represents the region of low MAF near a previously identified carcass weight QTL [15] represented by a yellow line. Click here for file
