# Supplementary figures and images for: High density linkage disequilibrium maps of chromosome 14 in Holstein and Angus cattle
Source: BMC Genet. 2008 Jul 8;9:45. doi: 10.1186/1471-2156-9-45 (PMC2478670; doi:10.1186/1471-2156-9-45)

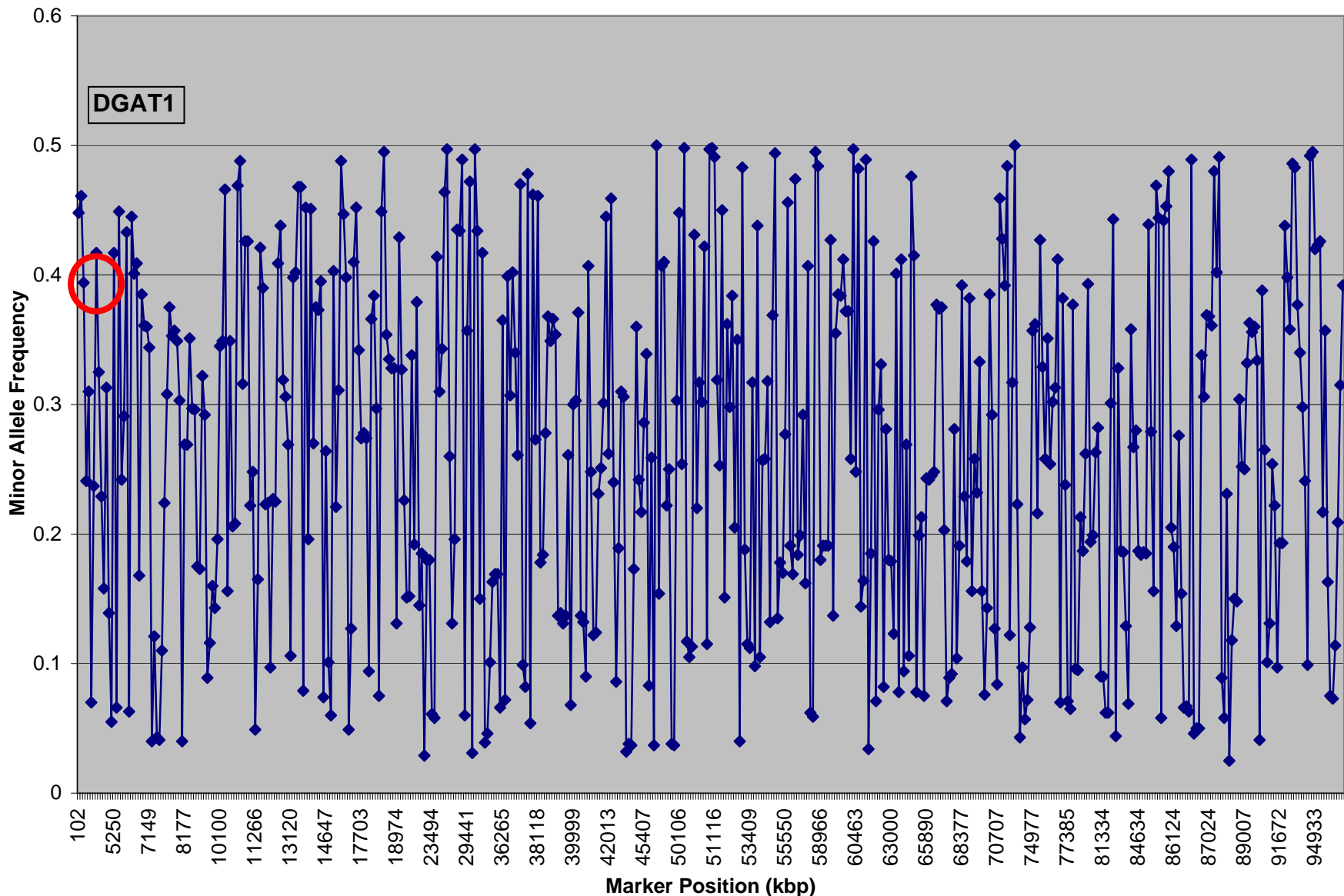

Supplement: Additional file 4 — Minor Allele Frequency (MAF) for 502 SNPs genotyped on Holstein. MAFs were plotted against marker positions (kbp) on bovine chromosome 14. Red circle depicts the position of acyl-CoA:diacylglycerol acyltransferase 1 (DGAT1). [file 1471-2156-9-45-S4.pdf]

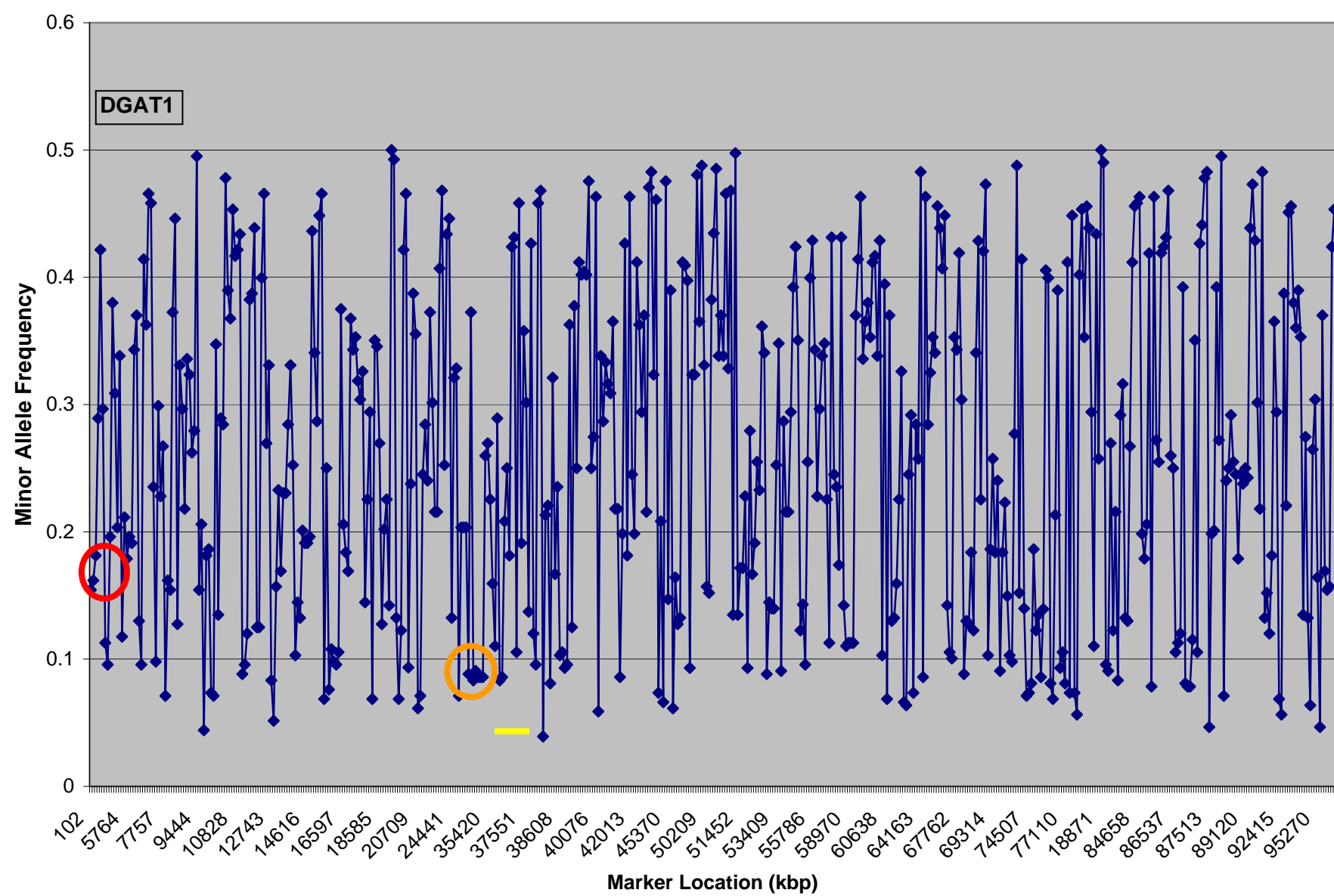

Supplement: Additional file 5 — Minor Allele Frequency (MAF) for 509 SNPs genotyped on Angus. MAFs were plotted against marker positions (kbp) on bovine chromosome 14. Red circle depicts the position of acyl-CoA:diacylglycerol acyltransferase 1 (DGAT1). Orange circle represents the region of low MAF near a previously identified carcass weight QTL [15] represented by a yellow line. [file 1471-2156-9-45-S5.pdf]
